# Patient Experience of Digitalized Follow-up of Antidepressant Treatment in Psychiatric Outpatient Care: Qualitative Analysis

**DOI:** 10.2196/48843

**Published:** 2023-10-11

**Authors:** Matilda Hamlin, Joacim Holmén, Elisabet Wentz, Harald Aiff, Lilas Ali, Steinn Steingrimsson

**Affiliations:** 1 Institute of Neuroscience and Physiology Sahlgrenska Academy University of Gothenburg Gothenburg Sweden; 2 Department of Psychiatry Sahlgrenska University Hospital Region Västra Götaland Gothenburg Sweden; 3 Institute of Health and Care Sciences Sahlgrenska Academy University of Gothenburg Gothenburg Sweden

**Keywords:** major depressive disorder, digital psychiatry, mobile app, adherence, antidepressant, antidepressants, depressive, depression, mHealth, mobile health, app, apps, application, applications, experience, interview, interviews, medication, prescribe, prescription, dose

## Abstract

**Background:**

Nonadherence to pharmaceutical antidepressant treatment is common among patients with depression. Digitalized follow-up (ie, self-monitoring systems through mobile apps) has been suggested as an effective adjunct to conventional antidepressant treatment to increase medical adherence, improve symptoms of depression, and reduce health care resource use.

**Objective:**

The aim of this study was to determine patients’ experience of digitalized follow-up using a mobile app as an adjunct to treatment concurrent with a new prescription, a change of antidepressant, or a dose increase.

**Methods:**

This was a qualitative, descriptive study. Patients at 2 psychiatric outpatient clinics were recruited at the time of changing antidepressant medication. After using a mobile app (either a commercial app or a public app) for 4-6 weeks with daily registrations of active data, such as medical intake and questions concerning general mental health status, individual semistructured interviews were conducted. Recorded data were transcribed and then analyzed using content analysis.

**Results:**

In total, 13 patients completed the study. The mean age was 35 (range 20-67) years, 8 (61.5%) were female, and all reported high digital literacy. Overall, the emerging themes indicated that the patients found the digital app to be a valuable adjunct to antidepressant treatment but with potential for improvement. Both user adherence and medical adherence were positively affected by a daily reminder and the app’s ease of use. User adherence was negatively affected by the severity of depression. The positive experience of visually presented data as graphs was a key finding, which was beneficial for self-awareness, the patient-physician relationship, and user adherence. Finally, the patients had mixed reactions to the app’s content and requested tailored content.

**Conclusions:**

The patients identified several factors addressing both medical adherence and user adherence to a digital app when using it for digitalized follow-up concurrent with the critical time related to changes in antidepressant medication. The findings highlight the need for rigorous evidence-based empirical studies to generate sustainable research results.

## Introduction

Depression is the most common mental disorder among adults and the second leading cause of the disease burden worldwide [1]. Standard pharmaceutical treatment is effective compared to a placebo among patients with major depressive disorder [2]. Even so, more than one-third of patients discontinue a new prescription within 3 months [2], and similar rates of discontinuation are also seen when increasing dosage, combining antidepressants, or changing antidepressants [3]. Nonadherence increases the risk of deterioration of psychiatric symptoms, with an increased number of emergency visits and hospitalizations, resulting in not only great suffering at an individual level but also an increased economic burden on the health care system [4,5].

Reasons for nonadherence to antidepressants are often multifactorial. Common factors are negative side effects or a lack of response to antidepressants, patient-related factors (eg, depressive symptoms themselves, psychiatric comorbidity, young age, and low socioeconomic status), and also environment-related factors (eg, shared decision-making and high availability and high continuity of specialized psychiatric care related to changes in medication) [6,7]. How patients handle the first weeks related to a newly prescribed antidepressant is a key factor, which is affected by guidance, information, and shared decision-making with the treating physician [8]. However, on-time high availability and high continuity between appointments to support, guide, and inform a patient are difficult factors to offer in a nonautomated manner. Novel and creative tools to increase self-care beyond the traditional health care setting may increase medical adherence to antidepressants at critical times.

The rapid implementation of digital psychiatry during the COVID-19 pandemic [9] will most likely continue to increase because of the potential postpandemic, pent-up demand for psychiatric care [10]. Beyond the broad implementation of digitalized health care and telecommunications, there is still potential for digitalized solutions with varying complexity and goals for health management to enhance psychiatric care, meaning that digital technology is used to fundamentally transform how health care is delivered and managed. A digital self-monitoring system can collect both active and passive data, whereby psychiatric care can be offered on demand, and it enables a patient to self-monitor the data collected, which can contribute to improved self-awareness [11]. However, it is important to develop sustainable systems suitable for both the health care provider and patient needs, while, at the same time, complying with existing regulations [12]. Self-monitored mobile apps as an adjunct to usual care can be an effective strategy in antidepressant treatment. Some studies report that a follow-up after changes in antidepressant treatment using mobile apps to report self-estimations of mood, sleep quality, and intake of medicine not only increases adherence to the pharmaceutical treatment [13,14] but also mitigates the severity of depression [15,16]. As an example, Corden et al [14] reported in a pilot study (N=11) how a mobile app together with a digital pill dispenser adjunct to initiation of psychopharmacological resulted in a mean medication adherence of 82%. The digital intervention included a reminder of medical intake in the absence of registration through the pill dispenser, weekly assessments of depression severity and medical side effects, graphical feedback of weekly assessments, and access to didactic lessons [14]. Furthermore, relatively easy interventions delivered via mobile phones, such as automatic reminders, can increase the user’s adherence to antidepressant medication [17], and this is well tolerated by patients [18]. However, other studies imply that reminders alone do not have a significant effect on adherence to medications and that additional efforts may be necessary to further increase adherence [19].

Engagement with and adherence to using a mobile app itself over time is a key factor that must be considered. Most people stop using an unguided mental health app 2 weeks after download [20]. Additionally, engagement may be difficult, specifically among patients with depression, given the symptomatology of a lack of motivation, a lack of interest, and impaired cognition, such as loss of memory [21]. Previous research has shown that factors promoting increased adherence to digital apps among patients with depression include reminder notifications, ease of use, and increased self-awareness. The latter can be achieved by continuously following and reviewing their own progress in an ecological context, which can increase motivation [21,22]. Furthermore, by creating an extra communication channel with a health care provider, the user’s engagement can increase and strengthen the therapeutic alliance between patient and clinician [22].

To summarize, digitalized follow-up is an innovative method to augment treatment at critical times among patients with depression. It may optimize health care resources by increasing medical adherence, improve symptoms of depression, and reduce health care resource use. However, several aspects must be considered before the dynamic and rapidly developing field of digitalized solutions in psychiatric care can be broadly implemented in clinical settings. The aim of our qualitative study is to elucidate patient experiences of using digitalized follow-up concurrently with a newly prescribed, increased dosage or a switch of antidepressant medication.

## Methods

### Study Design

This is a qualitative, descriptive study using individual semistructured interviews to collect data. Two different technical solutions were tested and interviews conducted thereafter. The method used for analyzing data is an exploratory qualitative content analysis design [23] with the intention to gain a deeper understanding of the topic and develop a clearer direction for future research.

### Participants and Settings

Eligible patients at 2 public health care psychiatric outpatient clinics located in southwest Sweden were asked to participate in the study either by a treating nurse or by a physician. No further advertisement of the study was conducted.

Patients were included if they had a new prescription, a change of antidepressant, or a dose increase. They also had to have mastered both spoken and written Swedish language. Exclusion criteria were patients in need of inpatient care due to depression, acute suicidality, or psychotic symptoms. All exclusion criteria were individually assessed by the patient’s treating specialist in psychiatry.

### Digital Intervention

Two different mobile apps were used to approach the study aim. One app was developed by a product company, and the other app used an existing e-service platform in public health care. The apps were tested and incorporated in the study design to minimize the risk of focusing on the experience of a specific mobile app and to, instead, broaden the research scope to examine the experience of using a mobile app as a method of follow-up.

Patients were assigned to use 1 of the 2 different mobile apps for 4-6 weeks prior to a semistructured follow-up interview. A research assistant, nurse, or treating physician trained in the use of the mobile apps instructed patients in how to use them. The health care provider was not obliged to monitor the patients’ recorded data, concerning which the patients were informed when enrolling in the study.

#### Commercial Mobile App

The first mobile app is called SENO (Medilevel), which is specifically designed to provide safe initiation of psychopharmacological treatment by offering daily remote monitoring and self-management [24]. The app was authorized and accepted by the IT department of the Västra Götalandsregionen (VGR) health care region. The log-in procedure includes 2-factor authentication with a 4-digit number received in a text message as a code. The app includes a pop-up reminder function at a self-chosen time.

Patients recorded data actively every day concerning intake of medicine, side effects according to a preset list of common side effects, and a grading of 3 questions concerning their general mental health status (energy, interest in things, and anxiety). The 3 questions were presented as 3 time plots, enabling a visual presentation of change over time. In addition, the Montgomery-Åsberg Depression Rating Scale—Self-Assessment (MADRS-S) was completed once weekly [25]. One-way text communication was possible if a health care provider wished to contact a patient through the mobile app.

#### Public Mobile App and E-Service

The second mobile app is incorporated into the existing and well-established e-service, 1177.se [26], which is used by all public health care providers in Sweden. The e-service can at present offer various features, such as medical advice, personal information about prescriptions or medical records, and a possibility to message your health care provider. The 1177.se app was specifically created for this study and replicated from the commercial app as closely as possible. The log-in procedure is with digital personal identification. A pop-up reminder function is possible but only at 10:00 A.M. Similar to SENO, patients recorded data actively every day concerning intake of medication, side effects according to a preset list of common side effects, and a grading of 3 questions concerning their general mental health status (energy, interest in things, and anxiety). The 3 questions were also presented as 3 time plots, and the app also enabled a time plot of weekly MADRS-S scores. Two-way text communication has already been incorporated in the e-service.

To summarize, the apps differed in following areas: The commercial app enables 1-way text communication for health care providers to contact the user, whereas the public app enables 2-way text communication. The time for a reminder is self-chosen in the commercial app, whereas it is at set at 10:00 A.M. in the public app. A time plot of MADRS-S scores is only available in the public app, and the font also differs between the 2 apps. Due to the nature of the study, no comparison was conducted between the 2 apps.

### Data Collection

Interviews were performed by either a female medical doctor and PhD student with minor experience in psychiatric care or a male nurse specialized in psychiatry. There was no established relationship between interviewers and patients prior to the study. The patients were interviewed individually after performing 4-6 weeks of digital intervention. In total, 3 interviews were conducted in person and the remaining 10 were conducted over the phone. This was primarily because of societal circumstances due to the COVID-19 pandemic.

The interview was semistructured whereby the patients answered prepared open-ended questions based on the study aim (Multimedia Appendix 1), with each question being followed by an open discussion. The questions were about patient experiences of the mobile app, such as “How did the app affect your medical adherence?” and “How did the app affect your appointment with your treating physician?” The mean duration of an interview was 22 (range 11-31) minutes.

### Data Analysis

A qualitative, inductive content analysis with a hypothesis-generating approach was used to find patterns and interpret meaningful content in the data [27]. Descriptive content analysis makes it possible to reach a high level of reliability through the adequacy of the analysis themes and draw valid conclusions from interpreting the data [28]. Due to the nature of the topic, patient statements were expected to be short and without latent content. The data were analyzed manually after being transcribed into text by the first author (MH). The analysis started with MH repeatedly reading all interview texts, forming a sense of the whole, and discussing initial interpretations of the whole with another author (LA). Thereafter, each interview text was divided by choosing meaning units, followed by condensed meaning units linked to the study aim. Next, a code for each condensed meaning unit was completed with a few words that represent the content. Finally, the different codes were distributed into themes and subthemes that represent a meaning found in several codes by MH and LA. All authors discussed the final themes and subthemes of the analysis.

### Ethical Considerations

An ethical committee at the VGR approved the study protocol (Dnr: 2020-01640). The study followed the Declaration of Helsinki principles with respect to research ethics. At a doctor’s appointment, patient consent was obtained for study participation after the patients were informed about the study both orally and in written form by a research assistant, treating physician, or treating nurse. All personal data were pseudonymized, and the audio data and transcribed data were stored in accordance with the Swedish General Data Protection Regulations.

## Results

### Participant Details

In total, 13 patients were interviewed between March 2021 and January 2023. The long recruitment period of nearly 2 years was partly related to the COVID-19 pandemic and partly due to reorganization at the outpatient psychiatric clinic. Two-thirds (n=9, 69.2%) of the patients used the commercial mobile app, and one-third (n=4, 30.8%) the public mobile app and e-service prior to the interviews. The difference between the type of app used was assumed not to affect the study aim; further participants were therefore not recruited to the public mobile app when data completion was fulfilled. The mean age of the patients was 35 (range 20-67) years. The demographic data of each patient is presented in Table 1. The most common reason for using the digital intervention was initiating new medical treatment either as monotherapy or as an add-on (n=6, 46.2%), followed by dose adjustment (n=5, 38.5%), while 1 (7.7%) patient used it for discontinuation and 1 (7.7%) for a switch between 2 medications. All patients were recruited from specialized psychiatric outpatient clinics and had undergone more than 1 previous medical treatment for depression.

We excluded 4 patients prior to the interview (n=3, 75%, did not answer multiple phone calls and text messages, and n=1, 25%, expressed in text an iatrogenic risk of using the app and participating in the study). None of the 13 (100%) patients who were included and completed the study expressed any deterioration in their mental illness due to the study.

**Table 1:** Characteristics of interviewed patients (N=13).

Participant	Gender	Age (years)	Digital intervention	Digital device	Digital literacy	Medication change
P1	Female	20	Commercial	iPhone	High	New prescription
P2	Female	26	Commercial	Android	High	Switch of medication
P3	Female	24	Commercial	iPhone	High	New prescription
P4	Male	27	Commercial	Android	High	New prescription
P5	Female	30	Commercial	Android	High	Change of dose
P6	Female	21	Commercial	iPhone	High	New prescription
P7	Female	32	Commercial	Android	High	New prescription
P8	Female	36	Commercial	iPhone	High	Change of dose
P9	Male	32	Commercial	NA	High	New prescription
P10	Female	61	Public	iPhone	High	Discontinuation
P11	Male	40	Public	iPhone	High	Change of dose
P12	Male	67	Public	iPhone	High	Change of dose
P13	Male	45	Public	Computer	High	Change of dose

### Main Themes

Four main themes emerged during data analysis: *adherence to using a mobile app*, *insight into one’s condition*, *support in a health care setting*, and *tailored content*. An overview of themes and subthemes is presented in Table 2, which is followed by a further description and illustration with quotations.

**Table 2:** Overview of themes and subthemes that emerged from the study data.

Themes	Subthemes
Adherence to using a mobile app	Daily registration Medical adherence Degree of mental illness
Insight into one’s condition	Questions leading to reflection Visual overview of patterns over time
Support in a health care setting	Increased understanding for the health care provider Evaluation of recorded data together
Tailored content	None

#### Adherence to Using a Mobile App

This theme describes the challenges of adhering to the mobile app over 4-6 weeks. It highlights the importance of creating a routine and how specific features, such as a systematic reminder and the time consumed for registration, can affect adherence. It also highlights that it can be challenging to adhere to and engage with a daily task beyond everyday activities or routines in a situation of a change in antidepressant treatment.

#### Daily Registration

The anticipated time to log in, register, and answer the daily questions was from 1 minute up to a few minutes. The main reason for nonregistration was forgetfulness. Therefore, to register every day, it is important to create a routine that fits in with daily life. Three key factors emerged as important if the patients were to adhere to registration every day: a daily reminder, a simple log-in procedure, and a non-time-consuming register of the daily questions. The majority of patients did include the importance of a daily reminder as a key function for daily adherence. As the preferred time of the day to register varied and was inconsistent between patients, and also for the same patient, many requested extra reminders that could vary in number and time each day. A few patients who used the public app did not receive the reminder due to technical issues, and they all maintained that it affected adherence negatively. The same patients also expressed the importance of being able to individually choose the time for a reminder.

Patients described that the log-in procedure with 2-way authentication via a text message was too complicated. The log-in procedure that had to be repeated when closing the app temporarily was frustrating. A few maintained that a simple log-in outweighed the possible benefits of increased data security. Many of those who used the commercial app emphasized that the same digital personal identification system used for the public app would have been a simpler and less time-consuming solution for log-in. Furthermore, the patients preferred that the recording of answers to the daily questions take only up to a maximum of a few minutes in order to maintain daily adherence. Several found the multiple-choice options and the grading of questions as positive and reported that free-text questions were too time-consuming and could therefore affect adherence negatively.

The majority of patients only reflected on the perspective of how to maintain adherence every day during the whole study period. However, 1 (7.7%) participant expressed:

To be honest, I think every day is a bit too often. I would say maybe once or twice a week. These types of symptoms don’t change very quickly, and one’s daily form fluctuates, so I understand if someone wants an average over time. However, I’m not sure if it would be as effective to fill in every day. That’s just my personal reflection. It could lead to worse adherence because it’s a bit tedious, and I know from experience that it can be challenging.

Patient 10 (P10), female, 61 years old

#### Medical Adherence

The first question in the app every day was “Have you taken your medication?,” with a response option of yes or no. A question to all patients therefore was “How has the use of the mobile app affected your medical adherence?” There were 4 (30.8%) patients who expressed a history of poor medical adherence for various reasons, such as forgetfulness and carelessness and also an active decision to not take a drug. A few of them expressed how an expectation when entering the study was to increase medical adherence. All these 4 (30.8%) patients showed increased medical adherence when using the mobile app, because of the notification to use the app and because the first question reminded them of their medication and whether they had or had not taken it. Even though medical adherence was not expressed as an issue among the other patients, an extra reminder was appreciated and not found to be unnecessary. No one reported worsened medical adherence due to the mobile app.

At times, I’ve been very careless with my medications...but I think this app has helped me...especially with this reminder to fill in the daily measurements, which also helps me remember my evening medication...there has been no carelessness during this period except for a few days when I genuinely forgot but that always happens.

P3, female, 24 years old

#### Degree of Mental Illness

Patients described adherence to the app as decreasing in situations in which they were feeling worse because of their depression. Using the app could feel like too big of an effort due to the loss of energy, and daily registration on the app, even taking only 1 or 2 minutes, could cause a setback in mood if one was having a bad day linked to their mental illness. Similarly, minor technical issues, such as a complicated log-in procedure or the patient getting logged out unexpectedly, could also cause frustration toward the app if the illness worsened. A few patients expressed that adherence to the app was good at first, but as their mental illness subsequently worsened over a few weeks, so too did the use of the app. The reasons included impaired memory and a feeling that nothing in the app would make them feel better or increase their motivation.

I know from my own issues that even the smallest things can feel difficult when you’re struggling. Having to schedule and do something every day when you don’t have the energy for anything can be overwhelming. Even though it’s the smallest thing...

P7, female, 32 years old

One further aspect reported by a patient was that she chose the timing of registration based on how she felt during the day. Since mental illness was perceived as way worse in the morning, she actively chose not to register at that time. Another patient reported how the lack of motivation toward using the app was constant since there was no improvement in depression throughout the whole study period.

#### Insight Into One’s Condition

This theme describes the experience of how the content can contribute to self-awareness and insight into one’s depression. The gain in self-awareness also had a perceived positive impact on the adherence to the app. Daily registration and additional content contributed to daily reflection and an understanding of their depression in several cases but did not necessarily lead to their own sense of improvement in the severity of depression.

#### Questions Leading to Reflection

To designate a few minutes every day and reflect on their own mental health status by answering the questions with respect to grading of mood, interest, and anxiety was a new experience for several of the patients. Many preferred to record during the evening as it made it possible to reflect on and summarize the day. The questions contributed to a reflection on not only *how* they were feeling on a specific day but also *why* they were feeling better or worse that day. To ask themselves how they were feeling and mapping it through the app made the patients feel good about themselves. It contributed to a feeling of doing something for their own sake and investing in something that would help them. The recording of answers had to be honest for it to be useful to them.

A participant experienced increased body awareness by asking themselves simple daily questions of mood, interest, and anxiety. However, another participant had not reflected at all on the recorded data, and yet another participant reported that daily recording on a bad day could have a reinforcing negative effect.

I have gained more, in a way, understanding or that I can see a connection with maybe things that have happened or how it has been, or so. It’s not strange that I’m completely exhausted when my mood goes one way or another.

P6, female, 21 years old

#### Visual Overview of Patterns Over Time

All patients who used the commercial app had a positive experience of the statistics function where the daily recordings were visualized as simple time series plots. The visual presentation of recorded data was described as interesting, meaningful, helpful, fun, valuable, supportive, and a way to solidify their depression. It was a valuable tool to help remember how they were feeling a week or several weeks ago and contributed to a concrete overview of patterns over the study period. The expectations to evaluate fluctuations in their depression over time increased motivation and adherence to the app, and several patients expressed how the statistics function was the reason they continued daily registration. Several requested more advanced diagrammatic functions, including additional self-selected variables.

The 4 (30.8%) patients who used the public app had difficulties in finding the statistics function and wished the function had lit up and been easier to find. However, they all maintained that such a visual function was something they had not experienced before and that it could be a potential help in their mental illness. In addition, 1 (7.7%) of the patients experienced the statistics function for the first time during the study interview. Interestingly, the self-perceived experience did not correspond to what the time series plot showed. When studying the time plot of MADRS-S scores, the patient also realized that he did not know how to interpret the score, even though he had filled out the form several times over previous years.

Let’s see, okay. Well, it has actually changed more than I thought when I look at it now. It actually has. It’s not how I experience it, but yes it has.

P13, male, 45 years old

#### Support in a Health Care Setting

This theme involves how the collected data were found to strengthen the patient-physician relationship. A potential use of the recorded data could be earlier contact with the patient and a strengthening of effort if the health care provider detects any abnormal recordings. The data recorded over time could also deepen understanding, contribute to a holistic view, and improve joint decision-making with a patient.

#### Increased Understanding for the Health Care Provider

Several patients expressed that the continuously recorded data were an improvement over usual treatment and could increase understanding by the health care provider. The statistics function was specifically emphasized as valuable to help the physician understand and obtain an overall picture of how depression had unfolded during the time between appointments. Eventual fluctuations of variables (mood, energy, and anxiety) could be presented visually without relying on the patient to remember, recall, and evaluate what information might be important for the treating physician. Furthermore, the physician’s understanding of depression could be enhanced even further if tailored content was possible, where patients could continuously add individual information that they considered valuable.

Furthermore, 1 (7.7%) patient retold a previous experience at a psychiatric outpatient clinic when her treating physician was temporarily replaced by someone else. The 2 physicians had different opinions and treatment recommendations based on the side effects that the patient experienced. The patient therefore thought that continuously recorded data over time could be valuable to physicians when taking over the treatment of a patient from another physician. Data beyond medical journals that are recorded by the patient themselves could help a new physician understand how the mental illness had fluctuated previously and what side effects there had been in relation to the treatment administered.

And for the doctor in this aspect as well, being able to see how it has fluctuated over time.

P11, male, 40 years old

#### Evaluation of Recorded Data Together

A digital communication with booking of appointments and video consultations was already a natural part of the outpatient clinics’ working approach. Even though there was already a way to message the outpatient clinics digitally (through 1177.se), the patients experienced difficulty in quickly getting in touch through this facility. Support and help from the outpatient clinics were sometimes not enough. The majority of patients believed that expanding the use of a mobile app as a standard when changing antidepressants could be a positive improvement, even though increased human contact would be the best alternative. Furthermore, those who used the public app expressed the benefits of the app being incorporated in 1177.se, mainly because all digitally provided health care would be gathered in 1 app and because they were already familiar with that app.

Patients expressed how data could be used as a potential marker of deterioration and a reason for earlier contact with a patient. The fact that all data were available and potentially evaluated by a health care provider in a timely manner increased adherence to the mobile app. Patients who used SENO expressed that a 1-way message function as an additional way to maintain contact with the outpatient clinics was positive. Patients considered the 1-way communication as a tool for the health care provider to potentially remind the patient to register or to quickly respond to abnormal data. However, there was an understanding and acceptance that quick 2-way communication was probably not possible, given the current lack of personnel and resources in psychiatric care.

Even though no one reported they had actually evaluated and discussed the recorded data together with their treating physician, it was still emphasized as a potential asset to the patient-physician relationship. If the physician notices any abnormal data between appointments, they can take the initiative to discuss the data without depending on the patient to retell information. Furthermore, to use the statistics function as support when discussing the patient’s mental illness could establish a more equal relationship, where the patient feels more involved and better understands decisions regarding treatment.

I think it’s very positive because when you meet or have contact, it would be good to have some flesh on the bones, simply put.

P12, male, 67 years old

#### Tailored Content

Tailored content means requesting individual adaptation of functions and content in the app to maximize its potential usefulness. There was mixed experience concerning the app’s content. The functions and content were in several cases too simple or too difficult and in some cases relatable or nonrelatable.

Most patients found both the content and graphics simple and clear. The simplicity contributed to the ease of use, with an acceptable use of time and with no difficulties in navigating the app’s features. The weekly recording of MADRS-S took a few additional minutes, but the patients had filled out this form several times before and were quite familiar with its contents. Furthermore, they experienced MADRS-S as an important and a comprehensive way to capture their depression.

Despite the ease of use, the majority of patients found the content to be too simple to improve their mental illness and said that a lack of in-depth and detailed content could affect motivation negatively. For the content to improve their mental illness, it was important to be able to relate to and fully understand the meaning of not only the questions asked as a whole but also the specific words used. As an example, 1 (7.7%) patient expressed difficulty in relating to the opposites “a lot of anxiety” and “little anxiety” when grading anxiety on a scale from 1 to 5, assuming that they have anxiety every day.

Additional content was requested, such as more information about side effects, the ability to grade the severity of each side effect, physical status, workload, sleep quality, and unexpected external events that affect mental status. Furthermore, many requested the additional content to be tailor-made, considering what are important and not important factors to a user when mapping their mental illness. For example, 1 (7.7%) patient expressed that all the collected data should be able to act as a visual self-written diary.

I would actually like to have it in a way that I could keep my own diary and track other factors that could affect my well-being with medications, such as how physically active I have been and how much I work. That’s what I mean when I say I want to make changes...yes. It would be really valuable to track things that one believes affect their well-being, like how often one has had psychotherapy, so that it’s not just about the medications…and maybe how one sleeps.

P10, female, 61 years old

## Discussion

### Principal Findings

The main finding of our study resulted in 4 themes that elucidate how patients at psychiatric outpatient clinics experienced digitalized follow-up using a mobile app concurrently with a new prescription, a change of antidepressant, or a dose increase. Adherence to both the mobile app and the antidepressant itself was positively affected by a daily reminder and relatively non-time-consuming daily registration. However, poor adherence to the app was attributed to the severity of mental illness in several cases. Positive experiences with the visual display of collected data in diagrams was a primary finding, which was beneficial for the patients’ self-awareness and reflection over their ongoing situation with mental illness. Furthermore, most patients believed that the mobile app, especially the visual display of data, can be a supplement to the patient-physician relationship and contribute to the physician’s understanding of their depression. However, none of the patients had actually discussed the content of the app with their treating physician. Finally, the patients had mixed reactions to the app’s content: some appreciated the ease of use and simplicity, but the majority requested more advanced and tailored content. There was no obvious deterioration in any patient’s mental illness linked to study participation.

The design and content of a mobile app can potentially affect the behavior of patients with depression. To understand the factors affecting engagement with and adherence to a mobile app is therefore a key factor for future implementation in clinical practice. The importance of both reminder notifications [29] and ease of use is in accordance with previous research [30]. However, even though a few patients preferred an easy log-in procedure at the expense of data security, trust in data security is reported as an important factor associated with a positive attitude toward digital health care [31]. Furthermore, studies have reported that several participants linked a higher severity of depression with reduced adherence to the mobile app [32,33]. A large-scale multinational study investigating the utility of smartphone apps to monitor depression by collecting both active and passive data showed that a higher severity of depression at baseline was associated with less contribution of both active and passive data to a mobile app [33]. The aspect that the severity of depression may affect the use of a mobile app is not surprising, given the core symptoms of depression. Elevated depressive symptoms are a common reason for nonattendance of scheduled appointments in health care; therefore, a mobile app with tailored support strategies might facilitate reestablishment of contact with a health care provider [34].

All patients expressed a willingness to help in the development of psychiatric care as a key factor for participation in the study; however, not many found it to be a potential tool to help themselves. An imbalance between intrinsic and extrinsic motivation has been previously reported as a barrier to user adherence, meaning that users must be informed and well aware of an app’s value for themselves and for health care providers [29].

The commercial app in this study was primarily designed to increase medical adherence to antidepressants, and interestingly, there were 4 patients using the commercial app who subjectively experienced increased medical adherence during the study. This is in accordance with previous smaller clinical trials, indicating that mobile apps with a daily reminder and collection of active data can increase medical adherence to antidepressants [14]. However, despite our study results, further large-scale clinical trials with objective measurements are still needed to fully understand how a mobile app can affect medical adherence.

A primary finding of the analysis was the positive experience of the feature with visual time series plots, how it contributed to self-awareness, and how it contributed to patient-physician communications. This is consistent with previous research [35,36]. First, a systematic review of experiences concerning data visualization through digital apps among patients with chronic neurological and mental health conditions reported how visualization of collected data can increase self-awareness, develop communication and understanding with the health care provider, and increase the user’s adherence to a mobile app [36]. Furthermore, Scheuer and Torous [35] recently investigated patient perceptions on how useful digitally collected data concerning their mental illness can be if presented visually in various graphs. Simple graphs were reported as being more useful (eg, survey scores); however, by having access to graphs with more complex data, the participants perceived an increased comfort in sharing them with a health care provider. In addition, 89% (25/28) of the participants in that study reported that visual graphs of both active and passive data would contribute to better communication with their treating physician. These results are substantiated by the emerging themes in our study, such as *insight into one’s* condition and *support in a health care setting*. If we were to add an extra finding, the majority of our patients found the graphs to be not only helpful in the patient-physician alliance but also as a tool for the physician to gain a deeper understanding and a holistic view of an individual’s depression. However, even though most patients experienced the app as a potential support in a health care setting, no one actually discussed the content of the app with the treating physician. It is reasonable to believe that this primarily depended on the nature of the study, where all patients were informed when enrolling in the study that the health care provider was not obliged to monitor the recorded data.

Depression is a heterogenous illness with diverse prominent symptoms and comorbidities, affecting people of all ages with varying digital literacy. Not surprisingly, a theme emerged of the patients desiring tailored content based on their own characteristics and preferences. Especially, there was an urge to tailor the content that was visually presented in graphs. A survey study [37] with open-ended questions investigated which outcome domains are important to patients (n=1912), informal caregivers (n=464), and health care providers (n=627) when evaluating antidepressant treatment. A large number of outcome domains (n=80) emerged from the study results, where many of those, such as daily functioning (eg, workload, active family life, or social life), are not measured in the questionnaires usually assessed in both clinical practice and clinical trials when evaluating antidepressant treatment. Furthermore, a systematic review mentioned previously [36] also reported the importance of customizing visually presented data and that this can be a key factor for long-term user adherence. Promising results of self-tailored content in internet-delivered cognitive behavioral therapy among patients with major depressive disorder have also been reported [38]. Overall, research indicates that customized treatment is important in all phases of treatment in this group.

To summarize, the results we found in our study agree in many ways with previous research concerning the digital use of mobile apps among patients with depression. However, the results contributed 2 additional perspectives we believe can be valuable in future research of using mobile apps as part of antidepressant treatment. First, our findings substantiate previous research that all data collected should be available and visually presented to both patient and physician. A well-informed patient with easy access to all collected data can increase both intrinsic and extrinsic motivation for adherence to treatment, even if the data are actively collected and rather simple, as in our study. The second perspective is that our study procedure reflects the dynamic and somewhat problematic nature of implementing digitalized solutions, such as mobile apps, in a clinical setting. The study aim was based on real-world observations with a supply-demand gap of health care at a vulnerable time of treatment among patients with depression. The patients highlighted several important areas of improvement, and their overall experience was that a digitalized follow-up has the potential to enhance and strengthen the delivery of health care. However, given the dynamic field of psychiatry with digitalized follow-up using mobile apps, the results may become outdated if modifications to the app are made explicitly based on participant experiences before conducting a controlled clinical trial. With endless new possibilities to collect data and create digital biomarkers through patient smartphones, how can we avoid a never-ending rat race in the research of digital psychiatry before implementation in a clinical setting? Studies of patient experiences are important, but likewise, studies of efficacy are important to reflect on how specific elements of digitalized solutions can affect patient behavior. Future research and implementation in clinical practice should rely on rigorous evidence-based empirical studies, considering the perspectives of multiple stakeholders with the aim to generate sustainable research results.

### Limitations

The interviews were conducted by 2 researchers separately (MH and JH) with different credentials and experiences in psychiatric care. The qualitative content analysis was thereafter only conducted by 1 researcher (MH), which can affect the intercoder credibility and trustworthiness of the results. However, the study results can be assumed to be trustworthy and credible, given that the analysis was continuously discussed together with a supervisor with great experience in qualitative research (LA) and subsequently discussed with all authors. Furthermore, since the study collected relatively insensitive data, the risk of recall bias was relatively low and the need for multiple researchers to conduct the analysis was therefore not crucial.

The fact that patients were recruited from specialized psychiatric outpatient clinics must be considered. Previous experiences with and therefore attitudes toward medication and adherence may vary wildly, which may also affect their usage and thoughts about the app. Furthermore, the majority of the patients expressed no previous problems to medication adherence, even though a specific goal of the digital intervention was to improve medical adherence, which could have affected study results.

One further limitation is that the study population was a relatively homogenous group with respect to age and gender. Furthermore, all patients reported high digital literacy, and potentially important information, such as the education level and residential status, was not collected. Lastly, no data were gathered on how many people declined to participate. All the factors mentioned here can affect the variation of the phenomenon and transferability. That is, the generalizability of our results is mainly relevant to patients willing to use digitalized follow-up, which is of importance since the broad implementation of such solutions may make health care harder to access for those who are unwilling to use digitalized follow-up. The implementation of digitalized solutions has a long way to go to supersede regular care, but the potential is enormous [39].

### Conclusions

Patients recruited from psychiatric outpatient clinics experienced digital follow-up using a self-monitoring mobile app as a valuable adjunct to antidepressant treatment at the time of medication change. A key finding that emerged from the derived themes was that easy access and visual presentation of collected data may improve medical adherence by promoting self-awareness and improved patient-physician relationship.

There are endless new possibilities to collect data and create digital biomarkers through mobile apps. Future research and implementation in clinical practice rely on rigorous evidence-based empirical studies considering the perspectives of multiple stakeholders, including patient experiences, with the aim of generating sustainable research results.

## Appendix

Multimedia Appendix 1 Interview guide.

## Abbreviations

MADRS-S: Montgomery-Åsberg Depression Rating Scale—Self-Assessment
VGR: Västra Götalandsregionen

## Data Availability

Due to local regulations, we are not able to share the data freely, but raw data can be obtained from the corresponding author upon reasonable request.
