# Supplementary material for: Patient Experience of Digitalized Follow-up of Antidepressant Treatment in Psychiatric Outpatient Care: Qualitative Analysis
Source: JMIR Ment Health. 2023 Oct 11;10:e48843. doi: 10.2196/48843 (PMC10600645; doi:10.2196/48843)
Supplement: Multimedia Appendix 1 [file mental_v10i1e48843_app1.docx]

Interview guide (original interview guide in Swedish)

- What were your thoughts when you were asked to join the study?
- If you were to think freely and decide the app’s content yourself, how would it be designed?
- How has the app affected your medication management? Well-being?
- How has the app helped you during appointments with your doctor? Discussions during appointments? Etc…
- How did you experience the apps content?
- How user-friendly did you experience the app?
- Can you describe the different parts of the app and your experience of the different parts?
- Which parts/elements did you use? Have any features been particularly valuable or less valuable for your well-being?
- How did you experience the security and safety due to using a digital app concerning your mental health?
- Can the study procedure be improved or done differently?
- Android, iPhone, other?
- How familiar are you with using smartphones?
